# Filamentous ascomycete genomes provide insights into Copia retrotransposon diversity in fungi

**DOI:** 10.1186/s12864-017-3795-2

**Published:** 2017-05-25

**Authors:** Tifenn Donnart, Mathieu Piednoël, Dominique Higuet, Éric Bonnivard

**Affiliations:** 1 Sorbonne Universités, UPMC Univ Paris 06, Univ Antilles, Univ Nice Sophia Antipolis, CNRS, Evolution Paris Seine - Institut de Biologie Paris Seine (EPS - IBPS), 75005 Paris, France; 2 Department of Plant Developmental Biology, Max Planck Institute for Plant Breeding Research, Carl-von-Linné Weg 10, D-50829 Cologne, Germany

**Keywords:** Comparative genomic, Fungi Copia retrotransposons, GalEa elements, Conserved Hairpin Site

## Abstract

**Background:**

The relative scarcity of Copia retrotransposons has been recently characterized in metazoans in comparison with the other superfamilies of LTR elements. Furthermore, Copia retrotransposons have often a particular dynamics that results in a highly predominant single clade of elements within a large host taxon, such as the GalEa-like retrotransposons in crustaceans. Taking advantage of the skyrocketing amount of genomic data available for fungi, we carried out the first large-scale comparative genomic analysis of the Copia clades in filamentous ascomycetes.

**Results:**

Screening 30 completely sequenced genomes allowed us to identify more than 2500 Copia copies with conserved LTR, which are distributed in 138 families. Their characterization revealed that fungal Copia diversity is much broader than previously thought with at least 27 clades, 23 of which likely correspond to new ones. While the Copia copy number is low in most species, the two clades GalEa and FunCo1 are widely distributed and highly dominate Copia content as they both account for 80% of the detected sequences.

**Conclusions:**

In Fungi, GalEa retrotransposons are restricted to Pezizomycotina in which they can make up an outstandingly high proportion of the genome (up to 10% in *Cenococcum geophilum*). At last, we revealed that fungal GalEa elements structurally differ from all other Copia elements with an absence of Primer Binding Site. These elements however harbor a Conserved Hairpin Site which is probably essential for their transposition.

**Electronic supplementary material:**

The online version of this article (doi:10.1186/s12864-017-3795-2) contains supplementary material, which is available to authorized users.

## Background

Transposable elements (TEs) have been identified in all eukaryotic species investigated so far and can make up large fractions of genomes [1]. Because of their huge effects on genome structure and dynamics, they are considered as one of the major sources of genetic variability in eukaryotes [2–5]. TEs are very diverse in terms of structural features, sequences and replication mechanisms [1, 6]. Based on their mode of transposition, TE families are classified into two classes [1, 5]: (i) transposons (DNA transposable elements or class II elements) replicate *via* a “cut and paste” mechanism with a DNA intermediate; (ii) retrotransposons (class I elements), a TE class specific to eukaryotes, replicate *via* a “copy and paste” mechanism, which relies on the reverse transcription of an RNA intermediate. Retrotransposons are subdivided in five major orders: LTR (Long Terminal Repeats) retrotransposons, LINEs (Long INterspersed Elements), SINEs (Short INterspersed Elements), Penelope and YR (tyrosine recombinase encoding) elements [1]. The LTR retrotransposons, LINEs and SINEs have been detected almost ubiquitously. In contrast, the Penelope elements are widely distributed among animal species, but seem to be rare among plants, protists and fungi [7] and the YR retrotransposons (e.g. DIRS1-like elements) have a patchy distribution in unikont species [8, 9]. TEs characteristics greatly impact their dynamics and success in the genomes. For example, while LTR elements make up the largest proportion of plant TEs, they are less predominant in animals. Thus, TEs distribution and abundance among genomes greatly depend on both the element type and the host taxon considered.

Within LTR retrotransposons three superfamilies (Gypsy, Copia and BEL/Pao) have been characterized to date. All of them encode usually two genes in a single or two open reading frames [10]: *gag* is the 5′-most gene and encodes proteins that form the virus-like particles; and the *pol* is located 3′ of *gag*, and encodes various enzymatic activities like an aspartic protease (PR), a reverse transcriptase (RT), a RNase H and a DDE-type integrase (INT) that are involved in the transposition mechanism. They are flanked by two direct LTRs (usually between 100 and 500 bp long), which encompass the promoter and regulatory regions. As such elements require a multi-compound machinery to be mobile, genomic copies easily become inactivated by mutations. TEs activity is also regulated by the effects of diverse silencing mechanisms that limit their expansion. In particular, in fungi diverse defense process inactivate repeated sequences, such as RIP (Repeat-Induced Point mutation) which promotes Cytosine to Thymine mutations or MIP (Methylation Induced Premeiotically) which only methylates TEs at C residues [11, 12]. Such mechanism appears frequent, as 48 out of the 49 tested fungi (subphylum Pezizomycotina) showed evidence of directional mutation [13].

Superfamilies of LTR retrotransposons display uneven relative abundances among eukaryotes [14, 15]. Whereas Gypsy and Copia elements are widely distributed among the genomes of plants, fungi and animals, no BEL/Pao elements have been identified in mammals or plants so far. In metazoans, the Gypsy elements are clearly the most abundant and BEL/Pao elements often appear more abundant than Copia retrotransposons, which are absent in one third of metazoan genomes [15, 16]. In fungi, the first transposable elements have been described in 1979 in yeast [17]. The presence of LTR retrotransposons in filamentous fungi was reported in 1993 [18], with Gypsy being the most abundant [19]. The percentage of fungi (77 species tested) found with Gypsy (87%) or Copia (77.92%) retrotransposons is quite high [20]. However, a genome-wide analysis of 45 diverse species of fungi [21] reveals that Copia elements are often scarce or absent, the copy number (a copy being defined by the authors as an element that still carries at least one coding domain) varies greatly, even between closely related species. Moreover, several examples show that Copia elements could have important impacts on genomes and genes. In *Phanerochaete chrysoporium*, Copia-like elements seem abundant and one element interrupts a putative member of the cytochrome P450 gene family [22]. In *Pleurotus ostreatus,* Copia copy number clearly varies between strains (145 copies, including 17 full-length, in PC15 *vs* 78 copies, 8 full-length in PC9); and if Gypsy were the main elements involved in the TE-mediated gene repression, at least one gene appear inactivated by a Copia insertion [23].

Because of their relative low copy number, little is known about the diversity and the predominance of different families of Copia elements in fungal genomes. Previous phylogenetic analyses of 70 Copia retrotransposons families have revealed two major branches [14, 21]. The branch 2 groups at least 13 widely distributed clades among eukaryotes, while the branch 1 comprises Ty (Pseudovirus) elements found in fungi together with four clades of CoDi-like elements from diatoms and the GalEa clade. Initially described in galatheids (Galatheid *Euminida* *a* *nnulosa* [24]), GalEa elements have been actually more successful among metazoan species than initially thought with some elements identified in Mollusca, Chordata, Cnidaria, Ctenophora, Echinoderma and Hemichordata [15]. Numerous GalEa sequences have been also identified from some microbial metagenomes collected during the Sargasso Sea surveys [25]. However, it remained impossible to determine which organisms they originated from. Subsequent studies also confirmed the presence of GalEa elements in Rhodophyta genomes (*Porphyra yezoensis* [26] and *Porphyridium cruentum* [15]).

In the present study, we took advantage of the skyrocketing amount of genomic data [27] to carry out the first large-scale comparative genomic analysis of the different Copia clades in fungi. We revealed that fungal Copia diversity is much broader than previously thought with at least 27 clades. After identifying for the first time some fungal GalEa elements, we wondered whether those elements could be highly predominant in comparison with other clades of Copia retrotransposons, a pattern we previously observed in Malacostraca [15]. To answer this question, we combined *de novo* and similarity-based *in silico* approaches to identify the Copia elements from 30 species. We also reveal that fungal GalEa elements structurally differ from all other Copia elements with an absence of Primer Binding Site (PBS). These elements however harbor a Conserved Hairpin Site which is probably essential in the transposition process.

## Results

### Copia retrotransposon identification

Thirty assembled fungi genomes were screened for Copia retrotransposons using LTRharvest. These genomes have been selected, independently of their phylogenic position and traits of life, according to the preliminary detection of GalEa element traces using BLAST searches on all assembled genomes available in the fungal genomics resource from MycoCosm database (Table 1). No complete element could be detected in *Pyrenophora teres*, *Colletotrichum higginsianum*, *Ophiostoma piceae* and *Daldinia eschscholzii*, because these genomes harbor only few copies with altered LTRs. In the 26 remaining species, we identified 2513 copies that can be clustered in 138 clusters using BlastClust [see Additional file 1 for details]. A cluster is hereby considered as a TE family. Sixteen species harbor less than 5 families, and only 3 species show a large diversity with more than 10 families (15 in *Talaromyces stipitatus*, 20 in *Pyrenophora tritici-repentis* and 31 in *Cenococcum geophilum*, respectively). The number of copies per families range from 1 to 559 copies and is small in average (median of 4 copies). We also identified 77 sequences that do not cluster with any other (orphan sequence). Surprisingly, 39 of these orphan sequences arose from a single species, *Erysiphe pisi*, which has an outstanding diversity. Since orphan sequences likely result from element degradation and correspond to non-functional copies, we did not consider them for the intra-species element diversity analysis.

**Table 1:** Number of Copia retrotransposons in fungal genomes

Class/Order	species	Sequences obtained with LTR Harvest				Sequences (>3Kb) obtained using RepeatMasker			
		GalEa	FunCo1	other Copia	GalEa/Copia	GalEa	FunCo1	other Copia	GalEa/Copia
Dothideomycetes									
Pleosporales	*Pyrenophora teres* ^pp^	0	0	0	ne	1	ne	ne	ne
Pleosporales	*Pyrenophora tritici-repentis* ^pp^	76	75	50	0.38	85	89	59	0.36
Incertae sedis	*Cenococcum geophilum* ^sb^	1246	184	192	0.77	2368	279	401	0.78
Eurotiomycetes									
Eurotiales	*Neosartorya fischeri* ^p^	1	2	9	0.08	1	2	11	0.07
Eurotiales	*Talaromyces aculeatus* ^s^	12	4	3	0.63	19	4	3	0.73
Eurotiales	*Talaromyces marneffei* ^p^	19	29	3	0.37	16	31	5	0.31
Eurotiales	*Talaromyces stipitatus* ^s^	40	30	14	0.48	51	35	27	0.45
Leotiomycetes									
Erysiphales	*Erysiphe pisi* ^pp^	13	5	34	0.25	23	7	53	0.28
Helotiales	*Botryotinia fuckeliana* ^pp^	3	2	0	ne	6	1	0	ne
Helotiales	*Chalara longipes* ^s^	4	0	8	0.33	11	0	21	0.34
Helotiales	*Meliniomyces bicolor* ^sb^	156	8	21	0.84	603	75	31	0.85
Helotiales	*Meliniomyces variabilis* ^sb^	3	11	10	0.13	5	25	31	0.08
Helotiales	*Sclerotinia sclerotiorum* ^pp^	1	10	8	0.05	1	12	8	0.05
Incertae sedis	*Oidiodendron maius* ^sb^	11	14	39	0.17	17	17	57	0.19
Sordariomycetes									
Glomerellales	*Colletotrichum fiorinae* ^pp^	0	0	1	ne	0	0	1	ne
Glomerellales	*Colletotrichum graminicola* ^pp^	19	0	35	0.35	151	0	53	0.74
Glomerellales	*Colletotrichum higginsianum* ^pp^	0	0	0	ne	1	ne	ne	ne
Glomerellales	*Verticillium albo-atrum* ^pp^	0	3	0	ne	1	3	0	ne
Glomerellales	*Verticillium dahliae* ^pp^	1	24	0	0.04	1	26	0	0.03
Hypocreales	*Beauveria bassiana* ^ip^	1	0	1	ne	2	0	1	ne
Hypocreales	*Cordyceps militaris* ^ip^	13	0	3	0.81	46	0	7	0.84
Hypocreales	*Metarhizium robertsii* ^ip^	0	0	3	ne	2	0	3	ne
Magnaporthales	*Gaeumannomyces graminis* ^pp^	0	5	2	ne	0	46	4	ne
Magnaporthales	*Magnaporthe oryzae* ^pp^	0	0	30	0.00	2	0	69	0.03
Magnaporthales	*Magnaporthe poae* ^pp^	0	0	2	ne	0	0	1	ne
Ophiostomatales	*Ophiostoma piceae* ^s^	0	0	0	ne	1	ne	ne	ne
Sordariales	*Chaetomium globosum* ^s^	5	7	6	0.28	7	9	6	0.32
Xylariales	*Daldinia eschschotzii* EC12^sb^	0	0	0	ne	2	ne	ne	ne
Xylariales	*Hypoxylon sp.* CO27-5^sb^	1	0	0	ne	4	ne	ne	ne
Xylariales	*Hypoxylon sp.* EC38^sb^	0	0	1	ne	1	0	1	ne

*ne* not estimated

^pp^plant pathogen, ^ip^insect pathogen, ^p^pathogen, ^sb^symbiont, ^s^saprotroph

### Copia retrotransposon diversity in fungi

**Fig. 1:**
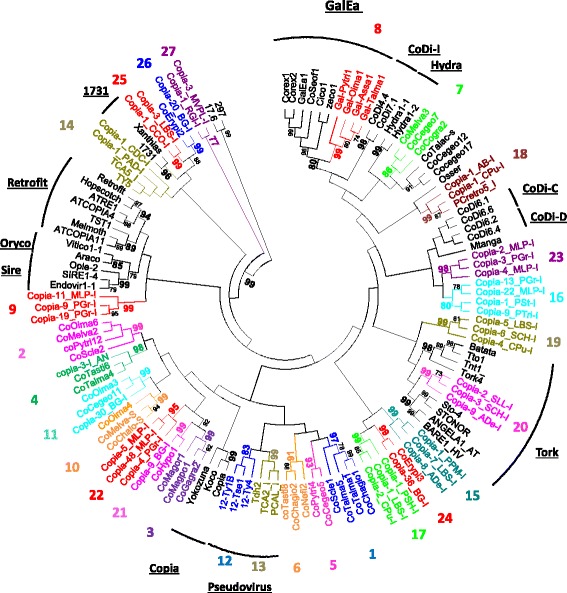
Phylogenetic relationships of Copia retrotransposons inferred from Neighbor-Joining analysis of RT amino acid sequences. The 27 FunCo (Fungal Copia) clades defined in Additional file 2 are indicated by their colored number and the previously defined Copia clades in the ‘Gypsy Database’ are underlined. Statistical support (>70%) comes from non-parametric bootstrapping using 100 replicates. Gypsy retrotransposon sequences (297 and 17.6) were used as outgroup

To infer the phylogenetic relationships of fungal Copia retrotransposons and estimate their diversity, we performed a phylogenetic analysis based on the amino acid RT domain of 412 elements that are representative of the newly identified families and the Copia sequences available in the fungal subset of the RepBase database [see phylogenetic tree in Additional file 2]. We defined 27 FunCo (Fungal Copia) clades based on the two following criteria: i) a clade comprises sequences from at least two species (large species-specific clades detected in *Blumeria graminis*, *Melampsora larici populina* and *Pucinia graminis* were thus excluded); and ii) a clade is supported by a bootstrap value higher than 70. To test whether FunCo clades belong to previously described Copia clades [28], a second phylogenetic tree has been built using few representative elements per FunCo clade and per reference clade of Copia previously reported in eukaryotes (Fig. 1). The resulting tree revealed that FunCo12 and FunCo13 correspond to the Ty (Pseudovirus) retrotransposons already identified in fungi, and that FunCo20 seems closely related to the Tork clade described in plant genomes. As expected, considering that the studied genomes have been selected according to the presence of GalEa elements, one clade, FunCo8, integrated GalEa elements from metazoans. The 23 remaining FunCo clades likely correspond to new Copia clades.

Eleven TEs clades appear endemic of Basidiomycota, 3 of Saccharromycotina and 13 of Pezizomycotina. Five of these Pezizomycotina-specific clades (FunCo4, 10, 21, 24 and 26) are quite rare, with less than 10 putatively functional sequences detected [see Additional file 1 and 2 for details]. On the contrary, the FunCo1 and GalEa (FunCo8) clades are widely distributed and highly dominate Copia content as they both account for ~70% of the described families (39 and 53 families, respectively).

### Copy number of Copia retrotransposons

We analyzed the distribution of the 2513 Copia copies detected using LTRharvest that targets potentially active elements as they harbor two conserved LTRs (Table 1 and Additional file 1). The copy number per genome ranges from 1 (*Hypoxylon spp.* and *Colletotrichum fiorinae*) to 1622 (*C. geophilum*) and is in average relatively low as two-third of the species harbor fewer than 25 copies. Five species show a moderate copy number (between 30 and 64) either due to a higher family diversity (e.g. *Oidiodendron maius*) or to a particularly large clade (e.g. the FunCo3 clade in *Magnaporthe oryzae*). Four species harbor more than 80 copies, with a particularly high copy number in *C. geophilum.* Such a high copy number is usually due to the GalEa and/or FunCo1 clades which account for 80% of the detected sequences (405 and 1615 copies, respectively).

We screened the 30 genomes using RepeatMasker (RM) with the FunCo elements previously identified. Only sequences longer than 3 kb (the smallest size obtained using LTRharvest) have been considered to reassess the copy number of Copia elements (Table 1). Whereas LTRharvest targets potentially complete elements, RM allows us to retrieve more altered copies with non-detectable LTRs. The presence of large GalEa fragments has been thus confirmed in 8 species that show negative results using LTRharvest. The three remaining species only show highly degenerated derivatives of GalEa elements (fragments smaller than 3 kb without conserved LTRs). As expected, we observed more copies with RM. However it is interesting to note that whatever the species or the Copia clade considered (FunCo1, GalEa or all other Copia), the copy number estimated using RM is most of the time approximately the double of LTRharvest copy number. However, both approaches give overall similar results in terms of relative abundance of the different Copia groups in the genomes as the two FunCo1 and GalEa clades clearly appear as predominant in RM estimations (~80%) as in LTRharvest results. In contrast, the two approaches greatly differ in copy number for three species with an increase by a factor 4 to 7. Differences are related to enrichment in elements from the FunCo1 clade (*Gaeumannomyces graminis*), from the GalEa clade (*Colletotrichum graminicola*) or from both clades (*Meliniomyces bicolor*).

### Genomic proportions of Copia clades

We estimated the genomic proportions of the different Copia clades (FunCo1, GalEa, and other Copia) considering all sequences obtained with RM (*i.e.* without minimum size, Table 2). Overall, Copia elements make up less than 1% of 14 of the 25 analyzed genomes and more than 2% of 6 genomes. Copia genomic proportion is likely influenced by host species phylogeny as they make up less than 0.5% in 8 Sordariomycetes species (out of 13 species) but more than 4% in the 2 Dothideomycetes genomes. Three species show an outstandingly high genomic proportion of Copia: *P. tritici-repentis* and *M. bicolor* (~5%, respectively), and *C. geophilum* (13%). For these last two species, this enrichment is mainly due to GalEa elements, which make up 5% of *M. bicolor* genome (82 Mb) and 10% of *C. geophilum* genome (177 Mb). Interestingly, while these two largest genomes have high copy number, there is no overall correlation between the abundance of Copia and the genome size.

**Table 2:** Copia retrotransposon abundance among fungal genomes

Class/Order	Species	Genome Size (Mb)	RIP-like^a^	Genomic proportion (%)^a^			
				GalEa	FunCo1	other Copia	GalEa/Copia
Dothideomycetes							
Pleosporales	*Pyrenophora teres*	33.58		0.15	ne	ne	ne
Pleosporales	*Pyrenophora tritici-repentis*	37.84	Yes [43]	1.57	2.00	1.17	0.33
Incertae sedis	*Cenococcum geophilum*	177.57	Probably	10.40	1.61	1.74	0.76
Eurotiomycetes							
Eurotiales	*Neosartorya fischeri*	32.55		0.03	0.06	0.22	0.08
Eurotiales	*Talaromyces aculeatus*	37.27		0.46	0.14	0.07	0.70
Eurotiales	*Talaromyces marneffei*	28.64		0.44	0.88	0.10	0.31
Eurotiales	*Talaromyces stipitatus*	35.69		1.27	1.10	0.41	0.46
Leotiomycetes							
Erysiphales	*Erysiphe pisi*	49.38	No [47]	0.48	0.11	0.97	0.31
Helotiales	*Botryotinia fuckeliana*	42.66		0.10	0.00	0.00	0.80
Helotiales	*Chalara longipes*	52.43		0.13	0.00	0.35	0.28
Helotiales	*Meliniomyces bicolor*	82.38		5.62	0.71	0.35	0.84
Helotiales	*Meliniomyces variabilis*	55.86		0.15	0.34	0.47	0.16
Helotiales	*Sclerotinia sclerotiorum*	38.33	Yes [73]	0.10	0.41	0.40	0.11
Incertae sedis	*Oidiodendron maius*	46.43	Yes [74]	0.25	0.26	1.12	0.15
Sordariomycetes							
Glomerellales	*Colletotrichum fiorinae*	50.04		0.06	0.00	0.01	0.82
Glomerellales	*Colletotrichum graminicola*	51.60	Yes [13]	2.52	0.00	0.95	0.73
Glomerellales	*Colletotrichum higginsianum*	49.08		0.07	ne	ne	ne
Glomerellales	*Verticillium albo-atrum*	32.83	Yes [75]	0.01	0.06	0.00	0.13
Glomerellales	*Verticillium dahliae*	33.83	Yes [75]	0.20	0.50	0.00	0.28
Hypocreales	*Beauveria bassiana*	33.69	No [76]	0.06	0.00	0.02	0.75
Hypocreales	*Cordyceps militaris*	32.27	Yes [54]	1.51	0.00	0.29	0.84
Hypocreales	*Metarhizium robertsii*	39.14	Yes [76]	0.03	0.00	0.14	0.17
Magnaporthales	*Gaeumannomyces graminis*	43.62		0.06	1.05	0.12	0.05
Magnaporthales	*Magnaporthe oryzae*	41.03	Yes [77]	0.02	0.00	2.38	0.01
Magnaporthales	*Magnaporthe poae*	39.50		0.05	0.00	0.07	0.41
Ophiostomatales	*Ophiostoma piceae*	32.84		0.03	ne	ne	ne
Sordariales	*Chaetomium globosum*	34.89	No [13]	0.13	0.18	0.10	0.32
Xylariales	*Daldinia eschscholzii* EC12	37.55		0.12	ne	ne	ne
Xylariales	*Hypoxylon sp.* CO27-5	46.59		0.09	ne	ne	ne
Xylariales	*Hypoxylon sp.* EC38	47.30		0.03	0.00	0.03	0.47

*ne* not estimated

^a^RIP-like mutation events already (Yes) or never (No) detected according to the reference mentioned; probably, according to our results

^b^Sequences obtained using RepeatMasker (unlimited size)

### Distribution of GalEa and FunCo1 elements among fungi

**Fig. 2:**
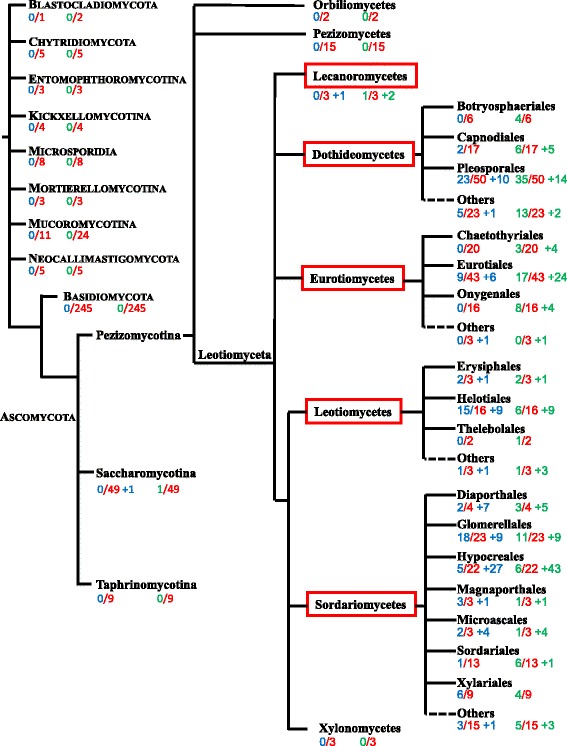
Distribution of fungal GalEa and FunCo1 elements. Species phylogeny was redrawn from MycoCosm [31]. The five Pezizomycotina classes in which GalEa and FunCo1 elements were detected are framed in red. In each group, the number of assembled genomes harboring GalEa (in blue) or FunCo1 (in green) sequences and the number of genomes analyzed (in red) are given. The number of other fungal species that have GalEa or FunCo1 retrotransposons according to BLAST searches on GenBank is given after the plus sign [See Additional file 3 for species details]

As GalEa and FunCo1 elements are two major Copia clades in the species tested, we wondered whether this feature could also be true in terms of distribution among fungi species. To test this, we screened the genomic or transcriptomic data available in MycoCosm and GenBank using few representative elements as queries. We detected GalEa and FunCo1 elements in 177 and 270 fungal species in total, respectively. Their presence is almost entirely restricted to one group of Ascomycota: the subphylum of Pezizomycotina (Fig. 2). In total, a third of the 317 Pezizomycotina genomes tested harbor GalEa elements [see Additional file 3 for details] and about 40% harbor FunCo1 elements. More precisely, all GalEa and FunCo1 elements were found in five classes belonging to Leotiomyceta (41 and 79 Dothideomycetes, 16 and 61 Eurotiomycetes, 1 and 3 Lecanoromycetes, 29 and 23 Leotiomycetes and 89 and 103 Sordariomycetes, respectively). Two of them correspond to Yeast-Like Symbionts from the two aphids *Nilaparvata lugens* and *Cerataphis brasiliensis*, which are thus classified within the Hypocreales [29, 30]. For the 3 remaining classes of Pezizomycotina (Orbiliomycetes, Pezizomycetes, Xylonomycetes), only few genomes are available to date [31]; too few to draw any reliable conclusion. In addition, few short GalEa-like sequences were detected in the whole-genome shotgun contigs of *Geotrichum candidum* (Saccharomycetales) and 2 FunCo1 copies were observed in the *Lipomyces starkeyi* genome (Saccharomycetales, [32]). However, no GalEa or FunCo1 element has been detected in the 57 other Ascomycota assembled genomes, so further investigations are requested to confirm the presence of these elements in Saccharromycotina. Similarly, no GalEa or FunCo1 element has been detected in Basidiomycota species (245 assembled genomes tested).

The distribution at a small phylogenetic scale is greatly influenced by the choice of the species for genome sequencing projects, mainly centered on fungi of interest, like pathogenic species. For example, GalEa and FunCo1 retrotransposons have been detected in only 5 and 6 of 22 Hypocreales assembled genomes, but they have been observed in 27 and 43 other Hypocreales species, respectively. However the large number of genomes studied emphasizes that GalEa and FunCo1 retrotransposons are widely distributed among Pezizomycotina (Fig. 2 and Additional file 3). Overall, they show a similar distribution among fungi classes and orders. However few differences can be noticed like the overrepresentation of FunCo1 elements in Dothideomycetes and Eurotiomycetes. This is particularly due to the distribution of elements that is also often uneven within an order. For example in Eurotiales, only few and short GalEa sequences have been observed in 4 of 24 *Aspergillus* and 2 of 13 *Penicillium* genomes, whereas all the 3 available *Talaromyces* genomes harbor numerous GalEa retrotransposons. In contrast, FunCo1 elements are well represented in 24 *Penicillium* species (Eurotiales) as well as in Onygenales order and 14 *Fusarium* species (Hypocreales) compared to GalEa elements.

### Fungal GalEa retrotransposons harbor an unusual ‘Primer Binding Site’

To describe the fungal GalEa retrotransposon features, we detailed the structure of 44 elements [Additional file 4] and compared the conserved DNA and amino acid motifs of 6 of them to those of 6 metazoan elements [Additional file 5]. The length of the fungal GalEa retrotransposons ranges from 5428 bp (Oima1 from *O. maius*) to 7018 bp (Cogra2 from *C. graminicola*), with an average length of 6100 bp. They appear thus larger than the GalEa elements previously described in metazoans (up to 4949 bp for CoRex1 [24]). They are however very similar to the other GalEa with : (i) LTRs bordered by 5’-TGT and 3’-CA with an average size of 235 bp (from 138 to 311 bp, excluding the outlier Cormil element that has 545 bp LTRs); (ii) a 5 bp Target Site Duplication as observed for Zeco1 in *D. rerio*; (iii) a large single ORF; and (iv) a great variability in the PolyPurine Tract signal. They also share several conserved motifs such as the HHCC and DD(35)E signatures of the Integrase, the DTG(C/A) signature of the protease and the ADxxTK sequence at the end of the RNase H domain, but slightly differ on some other conserved motifs [Additional file 5]. The zinc-finger (C(2)C(4)C(4)H) in the *gag* region is characteristic of fungal elements (C(2)C(4)H(4)C in metazoans) and the KSRLVI and QTDD motifs in the RT differ from the KARLVA and YVDD metazoan motifs. More contrastingly, the metazoan TRPDI motif at the beginning of the RNase H is substituted by a CQPEA motif.

**Fig. 3:**
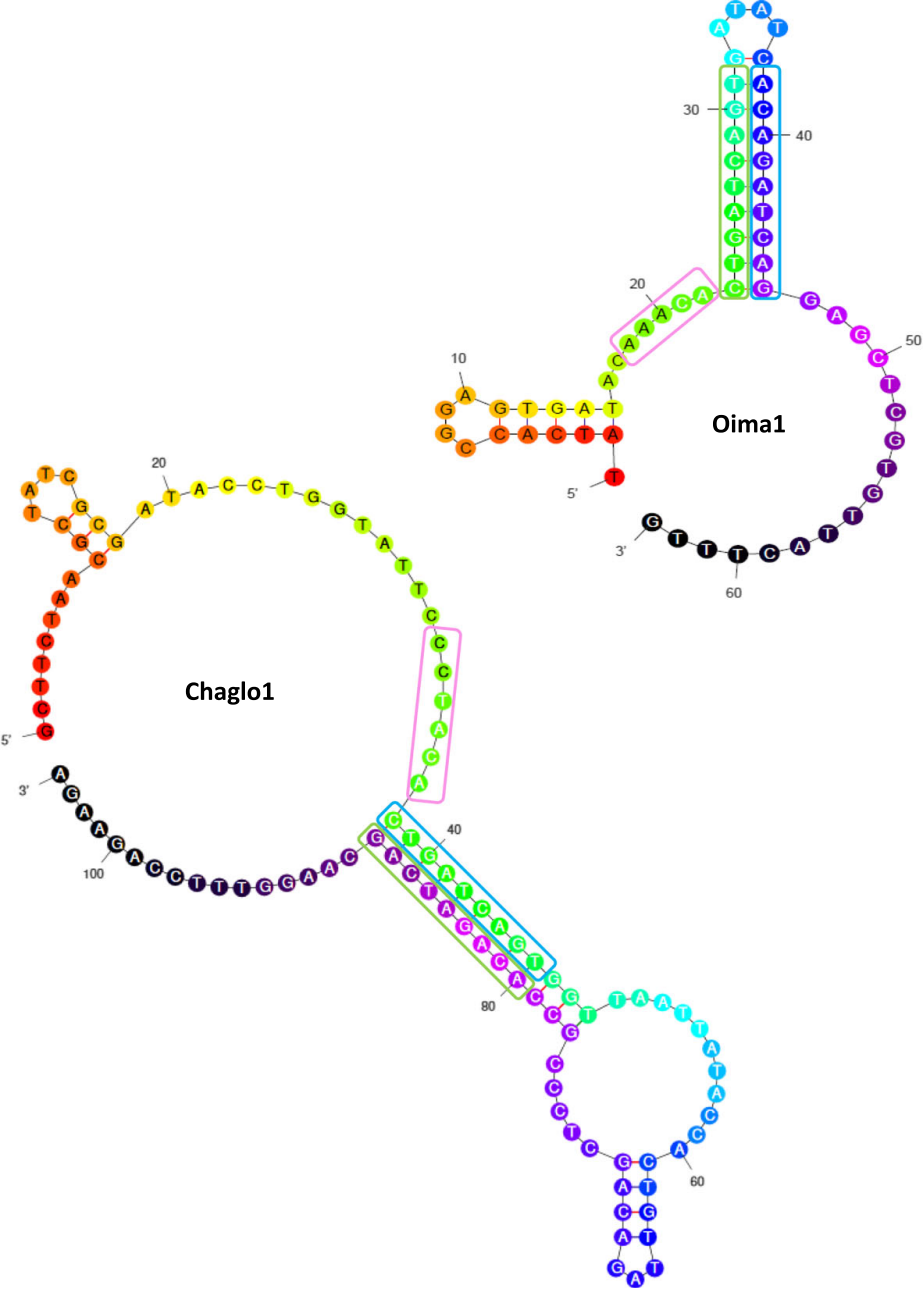
Hairpin secondary structure of the Conserved Hairpin Site (CHS) of fungal GalEa retrotransposons. Highlighted element features comprises the end of the LTRs (in pink), the conserved palindromic sequence CHSeq1 (in blue) and the complementary conserved sequence CHSeq2 (in green). Chaglo1: family from *Chaetomium globosum*; CoOima1: family from *Oidiodendron maius*

The major feature that distinguishes GalEa retrotransposons from fungi and metazoans is the Primer Binding Site. GalEa PBS was characterized in metazoans as a strictly conserved TGGTAGCAGAGC sequence, complementary to the 3’ end region of *D. melanogaster* tRNA^Met^ gene, located right after the end of the 5’ LTR [24]. In contrast, GalEa fungal elements do not show any putative PBS, while FunCo1 elements harbor a classical PBS (ATTAAGAGTCT), complementary to an internal region of *D. melanogaster* tRNA^Lys^ gene. They however harbor a conserved 9 bp sequence (called CHSeq1), which is palindromic when including the final A nucleotide of the 5’ LTR. In 85% of the families, this sequence is CTGATCAGT or CTAATTAGT [Additional file 4]. In the remaining families, different derivatives are observed, mainly originating from substitutions at the third and/or sixth nucleotide. Interestingly, the CHSeq1 is followed by another conserved 9 bp sequence (CHSeq2), distant from 12 bp to 41 bp. These two sequences are inverse complementary, except for a strictly conserved A/A mismatch between the nucleotide 7 from CHSeq1 and the nucleotide 3 from CHSeq2. Thus, they might allow the formation of a hairpin structure directly after the 5‘LTR (Fig. 3). We call this particular feature the Conserved Hairpin Site (CHS). Possibly, the CHS may be part of a more complex secondary structure with a larger hairpin that shows a bulge or an internal loop located at the LTR-CHS junction as predicted in 5 of 8 analyzed elements. Whereas only the CHSeq1 and the CHSeq2 are well conserved among species, the length of the sequence conserved between elements of the same species can be broader [Additional file 6]. Indeed, in five species a larger domain is surprisingly well conserved, even between distant elements that belong to different families. This ‘extended CHS’ measures from 33 to 62 bp and begins from the 5’ LTR end, upstream from the CHSeq1, and ends few nucleotides downstream from the CHSeq2 [Additional file 6]. Sometimes, elements from closely related species share the same ‘extended CHS’ as in 3 species of the *Talaromyces* genus.

## Discussion

GalEa retrotransposons were first described in decapods and are widely distributed among metazoans. Study of LTR retrotransposons in Malacostraca reveals two features: (1) Copia elements were not detected in numerous species and appear less diverse than Gypsy elements, which supports the Copia retrotransposon scarcity in metazoans already hypothesized in other taxa [16]; and (2) among Copia elements, the GalEa clade is highly predominant and show a species- or lineage-specific distribution that may be related to their “domino day spreading” dynamics [15]. This model is a branching process in which successive amplifications may interact positively. An analogy can be drawn between TEs dynamics and the propagation of domino falls. Like domino bricks following a restricted number of lines before toppling large structures, few active TEs copies are inherited prior to massive transposition events. Later on, the large domino structures allow the progression to the next structure *via* several paths, as the amplification of TEs increases the proportion of young active elements, which allow subsequent derived amplifications in some random lineages. Furthermore, the limited number of toppling dominoes between figures may facilitate the random breaking off of their progression along some paths. Similarly, evolutionary forces may drive the extinction of some elements within a lineage when elements are maintained too long at a low copy number. We thus wondered whether such dynamics can also be observed in a group closely related to metazoans within Opisthokonta, the fungi [33]. Fungal genomes are usually small (www.zbi.ee/fungal-genomesize/). For example, the 1C value varies from 0.007 pg (~6.8 Mb) to 3.12 pg (~3 000 Mb) (mean 0.04 pg, ~39 Mb) among 1254 Ascomycota species, with only three genomes larger than 1 pg (-978 Mb). Such genome sizes are clearly smaller than those observed in crustaceans whose 1C values are always larger than 1 pg [34].

Prevalence of Gypsy elements among LTR retrotransposons in fungi has been already revealed [21]. Focusing on GalEa elements, we did not study in-depth the distribution of all Copia retrotransposons in fungi. However our results are congruent with the previous observation and the highly variable copy number among genomes could explain Copia elements scarcity. In particular, Copia retrotransposons make up less than 0.5% of the genomes for half of the 30 tested species (selected on the presence of GalEa elements) and were not detected using LTRharvest in a third of them. So, considering all inherent detection limits related to the PCR approach used on crustaceans, the overall distribution of Copia elements among crustaceans or Pezizomycotina spp. appear similar.

Considering the prevalence of GalEa elements among Copia retrotransposons, Pezizomycotina results clearly differ from those obtained in crustaceans. GalEa elements do not appear predominant in any Pezizomycotina class or order, even if they often make up one of the most abundant Copia retrotransposon clade in the tested genomes. For example, they account for at least one third of Copia retrotransposons in 17 of 25 studied genomes. Interestingly, genomes where GalEa elements have been successful are dispersed throughout the species phylogeny (Table 2), even if they are sometimes widely distributed in some groups such as Helotiales and *Talaromyces* genus (Eurotiales). For the latter, the three complete genomes available have several dozens of large copies and several elements are detected in two other species. This contrasts with the other 38 Eurotiales assembled genomes that have none or only a single short GalEa copy [Additional file 3]. If confirmed, such a feature may help to differentiate *Talaromyces* from *Penicillium* species, two closely related genera that are difficult to discriminate [35]. Likewise, species from the genera *Pyrenophora* or *Colletotrichum* display significant differences in their number of GalEa elements. It suggests sudden amplification in copy number likely resulting from recent bursts of transposition in only few species. At last, *M. bicolor* and *C. geophilum* are also distinctive because of their very high GalEa copy number. These elements mostly clustered into 1 and 3 families, respectively, which might also reflect some transposition bursts. Future analyses of additional *Cenococcum* and *Meliniomyces* genomes are however requested to confirm these patterns.

The main reason why GalEa retrotransposons does not prevail among Copia elements is probably the presence of the second “abundant” Copia clade, FunCo1, also restricted to Pezizomycotina. If one ignores FunCo1, then GalEa elements are predominant in more than 60% of the species and together these two clades predominate in 80% of them. The species- or lineage-specific distribution of Copia, their variability in copy number and the small number of Copia families obtained per species [Additional file 1] remain congruent with the “domino days spreading” dynamics model. As fungi correspond to a much wider taxon than crustaceans, this model does not involve here a single but few major Copia clades: such as GalEa, FunCo1, and to a lesser extent FunCo2 and FunCo3.

Among GalEa retrotransposons, fungi elements are clearly distinct. Phylogenetic relationships inferred from analysis of the RT/RNaseH amino acid sequences of 42 elements from 27 diverse fungal species and 52 elements from 4 Rhodophyta and 33 metazoan species reveal that fungal GalEa elements cluster into a highly supported monophyletic group (bootstrap value of 98; Additional file 7). The single origin of fungal GalEa is supported by a singular feature, the lack of PBS, replaced by a CHS. We suspect that this CHS is functional because of the very high conservation of the two reverse complementary sequences (conservation even higher than in LTRs). The presence of extended CHS shared between elements of different families highlights the particular selective pressure acting on this non-coding region. Because its location coincides with the expected PBS location, we hypothesize that CHS plays the role of the PBS in the transposition cycle. However, to our knowledge, no previously described models fit to the CHS. Reverse transcription of most retrovirus and LTR retrotransposons required cellular tRNAs to serve as primers of minus-strand strong-stop DNA synthesis [36]. In contrast, few LTR retrotransposons developed other strategies to ensure reverse transcription initiation. For example, Tf1 from *Schizosaccharomyces pombe* uses a self-priming mechanism to initiate synthesis of reverse transcript instead of a primer derived from tRNAs [37]. Similarly, the initiation of reverse transcription of Rous sarcoma virus requires the formation of an additional RNA stem-loop structure [38]. However, the Tf1 mechanism requires a perfect complementarity between its PBS and the first nucleotides of the element mRNA and we were never able to detect any U5-inverted repeat sequence complementary to the CHS. The hairpin structure is fundamental for Tf1 transposition but the DNA sequence comprised in the loop doesn’t affect the function of transcription initiation [37]. This is probably the reason why the DNA sequence in the loop of the CHS is also the less conserved. At last, the PBS of Tf1 and of the Rous sarcoma virus are not palindromic. Even if it is difficult to predict which role it plays, the conservation of the palindromic structure of the Sequence1 in all the fungal GalEa elements we analyzed should draw our attention. The dimer initiation site (or DIS) is another particular structure that implies reverse transcription which combines a palindromic sequence and loop described in many retroviruses [39, 40]. However, this hairpin structure is observed in addition to the PBS and the palindromic sequence is localized within the loop.

GalEa retrotransposons are now described in numerous metazoan and Pezizomycotina species. Phylogenetic analyses [Additional file 7] showed that elements from these species form two monophyletic groups. Altogether it suggests that GalEa elements are ancient and were the most likely already present in the last common ancestor of Opisthokonta. They would have then diverged after the fungi-metazoans radiation. Thereafter, GalEa elements persist in various groups of metazoans and almost only in Pezizomycotina in fungi. The loss of GalEa retrotransposons in several large fungal groups (e.g. Basidiomycota according to the 174 genomes tested) or in some Pezizomycotina orders could be facilitated by the usually low copy number observed in the genomes, which suggests that the element activity is relatively low. This is also consistent with the small genome size of these organisms (Table 2) and the “domino days spreading” dynamics [15].

Comparative genomic studies of the abundance of repetitive sequences between distant species require reliable estimation. This is particularly an issue for transposable elements as even the copy or sequence concepts may correspond to different definitions according to the authors: complete or full-length elements, truncated elements, coding domains, derivatives such as solo LTRs, etc. This is why we combined different approaches that allowed us retrieving different types of sequences. The copies detected with LTRharvest are potentially functional, because their LTRs remain well-conserved in structure and in sequence. All of them were retrieved using RM, in addition to other more altered large (>3 kb) copies. We then based our estimations of genomic proportions on RM results regardless of the detected sequence sizes.

To compare the dynamics and the conservation of GalEa elements to all other Copia retrotransposons, we estimated the genomic proportions of GalEa among Copia elements based on the LTRharvest results or RM results (with or without element length threshold). The ratio based on LTRharvest results estimates the proportion of 'active' GalEa elements compared to the other active Copia elements. In contrast, the two RM ratios, compared to the LTRharvest estimator, reveals whether the GalEa elements are more or less (RM ratio < LTRharvest ratio or RM ratio > LTRharvest ratio, respectively) conserved than the other Copia retrotransposons. Interestingly, there are very good correlations and all estimators give similar results [Additional file 8], meaning that Copia retrotransposons of the different clades from any Pezizomycotina species are overall subjected to the same selective pressures and accumulate mutations at the same rate. Besides, the correlation between RM estimators [Additional file 8A] show that large copies usually make up most of Copia fractions in these genomes. Moreover, the correlation between large copies estimators [Additional file 8B] suggests that the loss of LTRs is independent of the Copia clade considered. At last, the analysis of such estimators allows to quickly pinpoint the few genomes where the GalEa or other Copia elements strongly differ from the elements detected in the other genomes in terms of selective pressure, copy number, element size, etc. as they deviate from the regression line. For example, comparison of the GalEa/Copia ratios estimated either from the copy number detected using LTRharvest or the genomic proportions derived from RM [Additional file 8C] highlights major differences in two species. In *C. graminicola*, this difference (35% *vs.* 73%) clearly results from a significant high number of large GalEa copies which could not be detected with LTRharvest. In *Verticillium dahliae*, this difference (4% *vs.* 28%) is due to a very high number of deleted GalEa sequences of 1 to 3 kb size, which strongly increase the proportion of GalEa among Copia.

Our findings also underline the importance of not restricting comparative genomics of TEs only at the level of superfamilies (Gypsy, Copia, BEL/Pao …). The identification of two major clades shows that the impact of TEs on the genome can vary greatly depending on the clade and host considered. Thus, the comparative study at the level of clades may provide new knowledge on the evolution of TEs (e.g. the selection of an unusual structure such as the CHS of GalEa). While we were mainly interested in studying evolution and distribution of GalEa elements in fungi, we largely extended the overall diversity of fungal Copia elements revealing 24 new clades. It would be interesting to establish to what extent the study of other genomes, such as those having FunCo1 elements and/or those of species outside Pezizomycotina, would increase the observed diversity. Moreover, such a study will allow comparison of the distribution of GalEa to other Copia clade within fungi, and testing whether clades apparently underrepresented are actually more frequent in other species groups. Such analysis of Copia retrotransposons would be facilitated by precise annotations of TEs within genomes. Reciprocally such genome annotation is now easier with a proper precise classification of the 27 fungal Copia clades.

## Conclusions

In this study we carry out the first large-scale comparative genomic analysis of the different Copia retrotransposon clades in fungi. These elements appear more diverse than previously thought, with 23 new clades characterized. Two of them account together for 80% of the detected sequences and can make up an outstandingly high proportion of the genome. These results support the “domino day spreading” dynamics model for Copia element previously described on crustaceans, which involves that only few Copia clades will highly dominate Copia content in a host taxa. One predominant fungal clade corresponds to GalEa elements, suggesting that these elements were the most likely already present in the last common ancestor of Opisthokonta. Interestingly, fungal GalEa elements clearly differ from metazoan GalEa elements as they form a distinct monophyletic group and as they are structurally singular with an absence of a classical Primer Binding Site. These elements instead harbor a Conserved Hairpin Site which is probably essential in their transposition process.

## Methods

### Preliminary detection of GalEa and FunCo1 elements in fungal genomes

To determine fungi species that potentially harbor GalEa or FunCo1 elements, we performed tBLASTn and tBLASTx [41] analyses on all assembled genomes available in the fungal genomics resource from MycoCosm [31]. Amino acid RT/RNaseH domains of GalEa elements from three different phyla have been used as queries: one previously characterized in Metazoa (GalEa1, DQ913005.1); one from the fungi *Metarhizium anisopliae* (XP_007817138.2) and from one from the Rhodophyta *Grateloupia lanceola* (HM767188.1). For FunCo1 we used the RT/RNaseH domains of an element from *Sclerotinia sclerotiorum* (Helotiales, CP017828.1). To discriminate the sequences that could belong to other Copia clades, we also used the *Copia* element from *Drosophila melanogaster* (X02599.1) as a query.

### Data mining for Copia elements in fungal genomes

Assembled genomes [42–60] were downloaded from the Joint Genome Institute Genome Portal [61] and the Broad Institute of MIT and Harvard (https://www.broadinstitute.org/). We first *de novo* isolated all potential LTR retrotransposon sequences using LTRharvest [62] based on the detection of two conserved LTRs and the following parameters: LTR length ranging from 100 to 1000 bp, distance between LTRs ranging from 3000 and 11000 bp and sequence identity between LTRs higher than 80%. To discriminate Copia elements from the other LTR retrotransposons or from artifactual sequences, we performed BLASTx similarity-searches on a custom database comprising RT/RNaseH amino-acid sequences from 116 Gypsy, 122 BEL/Pao, and 164 Copia retrotransposons (including 97 GalEa elements). This database encompasses sequences from de Gypsy database, appended with published BEL/Pao [16], Copia and Gypsy [15, 24] sequences.

The resulting datasets of Copia nucleotide sequences (including LTR parts) from each genome were separately clustered using BLASTclust (http://toolkit.tuebingen.mpg.de). Because the clustering results highly depends on the complexity of the detected sequences (e.g. nested elements), we empirically estimated the most appropriate values of BLASTclust parameters for each genome. Clusters were first defined using 70% percent identity threshold with 50% sequence length to be covered. Then, remaining sequences were tested to belong to one of these clusters using 90% percent identity with only 10% sequence length. This makes possible to retrieve sequences that clearly belong to a cluster but are greatly altered by large insertion or multiple gaps, for example. The sequences from each cluster were then aligned with the E-INS-i iterative refinement configuration of MAFFT version 7 [63], and were manually curated to remove all copy-specific insertions larger than 20 bp. Indeed, individual copies may be corrupted by insertion of various genomic sequences such as microsatellites or other transposable elements. Such an approach allowed us in particular to filter out chimeric structures, which comprise a mix of transposable element domains bordered by two conserved LTRs that have been described in fungi genomes [21]. Even if they comprise a Copia sequence, such peculiar structures would have biased the estimation of abundance of Copia elements among the genomes using similarity-searches. We finally checked that all the curated copies from a cluster share at least 80% of DNA sequence identity considering the complete sequence, a threshold often used to define transposable element families. Conversely, when the elements from two clusters share more than 80% sequence identity, the clusters have been merged into a single family. When a single GalEa sequence was detected in a species, it has been considered by default as a representative of a family.

At last, the genomes have been screened to recover additional Copia related sequences, especially some putative false negatives from LTRharvest and some shorter element derivatives. For that purpose, we used RepeatMasker [64] (options -nolow -no_is -pa 8 –frag 380000 -div 20) and a custom repeat database for each genome. This database comprises all curated Copia sequences identified in the studied genome (using LTRharvest or in the preliminary tBLASTn analyses).

### Distribution of GalEa and FunCo1 elements in fungi

To describe the distribution of GalEa and FunCo1 elements in fungi, we performed tBLASTn analyses (E-values 1e-75, Query cover > 50%) on all genomic or transcriptomic databases provided by the National Center for Biotechnology Information [65] using the same DNA sequences used for the preliminary detection of GalEa and FunCo1 elements. To determine whether the newly identified elements actually belong to the GalEa clade, we used two complementary approaches: sequences covering the RT/RNaseH domains were included into phylogenic analyses whereas the remaining sequences were classified using similarity searches using BLAST on the Gypsy Database, which includes clearly annotated and classified reference elements that represent all the different clades LTR-retrotransposons. In the latter case, an element was assigned to the GalEa clade when: (i) the five best hits correspond to the five referenced elements from this clade in the database; and (ii) the difference between the best E-values obtained with GalEa and other reference elements is higher than 1E^-10^.

### Phylogenetic analyses

Several phylogenetic analyses were performed on amino acid sequences corresponding to the RT or RT/RNaseH domains of the newly characterized Copia sequences, reference fungal Copia elements from RepBase or Gypsy Database, and/or previously identified GalEa retrotransposons [15, 24]. Boundaries of RT/RNaseH domains have been predicted using rpstBLASTx (E-value 10^-5^) and the pfam07727 profile, and the ‘ADxxTK’ conserved motif at the 3’ end of the RNaseH. DNA sequences were translated using a custom made script, manually curated and the longest representative of each family was selected. If sequences were corrupted by too many frameshifts and indels, we tried to manually reconstruct the protein sequences from the 6 frame translation obtained on http://bio.lundberg.gu.se/edu/translat.html. This especially afford to translate ripped sequences (AT-content >70%).

Multiple alignments of protein sequences were performed using MAFFT. After manual curation of the alignments, phylogenetic analyses were conducted using Neighbor Joining [66] and the pairwise deletion option of the MEGA5.2 software [67]. The best-fit model, the JTT model [68] with gamma distribution, was selected with Topali2.3 software [69] and support for individual groups was evaluated with non-parametric bootstrapping [70] using 100 replicates.

Most of the orphan sequences were discarded from phylogenetic analyses. To test whether they could belong to another family described in another species, we performed BLASTx (E-values 1e-150 on at least 300 amino acids) on the protein database of clustered Copia sequences. Some orphans could thus *a posteriori* be assigned to fungal Copia clade described in our phylogenetic analyses.

### In-depth characterization of fungal GalEa elements

The structure of newly identified GalEa elements has been in-depth characterized. In particular, the boundaries of the LTRs were manually analyzed, most of the times using a local alignment of all the copies belonging to a single family. ORFs were predicted using ORF Finder (https://www.ncbi.nlm.nih.gov/orffinder/) and the putative PPTs were assigned using LTR_finder [71]. Analyses of the nucleic acid folding and hybridization predictions on the CHS were performed on the Mfold web server [72]. At last, we identified the amino-acid sequences corresponding to the protein conserved motifs that have been previously described in GalEa elements [24].

## Additional files

Additional file 1: Copy number and genomic proportions of the clades and families of Copia retrotransposons detected in Pezizomycotina genomes. (XLSX 27 kb)

Additional file 2: Phylogenetic relationships among fungal Copia families. Neighbor-Joining analysis of RT amino acid sequences of representative Copia families isolated with LTRharvest and all fungal Copia sequences available in RepBase. The 27 FunCo (Fungal Copia) clades are represented by their number in bold color. Statistical support (>70%) comes from non-parametric bootstrapping using 100 replicates. (PPTX 573 kb)

Additional file 3: Distribution of GalEa retrotransposons among fungi according to type of data analyzed. Classification was redrawn from MycoCosm. The 30 assembled genomes analyzed in this study are highlights in green and assembled genomes apparently devoid of GalEa elements in red. Species in which the presence of GalEa elements was revealed by BLAST searches on partial genomic or transcriptomic data are indicated in orange with the accession number of the best hit. (XLSX 38 kb)

Additional file 4: Annotation of fungal GalEa retrotransposons. The copy number corresponds to the number of elements returned by LTRharvest. The two most frequent sequences of the CHS (Conserved Hairpin Site) CHSeq1 are given in green and blue, and their different observed derivatives are shown in light blue. Mismatches between the sequences CHSeq1 and CHSeq2 are indicated in red, including the strictly conserved A/A mismatch at the third position of CHSeq2 (in red bold). The Interval corresponds to the distance between the two reverse complementary CHS sequences. The GC content was estimated on all entire sequences of each family. (XLSX 20 kb)

Additional file 5: Comparison between structural features of 7 fungal and 6 metazoan GalEa retrotransposons. Features from metazoan elements were described in Terrat et al. (2008) and Piednoël et al. (2013). (XLSX 28 kb)

Additional file 6: Charaterization of the extended Conserved Hairpin Site. (A) Local alignment of Tasti2, Tasti4 and Tasti5 sequences from *Talaromyces stipitatus* showing the different regions of the extended Conserved Hairpin Site (CHS). (B) Families in which different extended CHS were observed. For each extended CHS observed, the corresponding size (in bp) of its variable regions is given. (PPTX 126 kb)

Additional file 7: Phylogenetic relationships among GalEa retrotransposons. Neighbor-Joining analysis of RT/RNaseH amino acid sequences of GalEa elements and representative Copia clades previously defined in the Gypsy Database. Statistical support comes from non-parametric bootstrapping using 100 replicates. (PDF 178 kb)

Additional file 8: Comparison of methods used to estimate the proportion of GalEa elements among fungal Copia retrotransposons. We compared in pairs the three estimations of GalEa proportions among Copia based on the number of copies detected with LTRharvest, the number of large copies detected with RepeatMasker, or the genomic proportions derived from RepeatMasker for the 17 genomes that harbor at least 10 Copia sequences detected with LTRharvest. (PDF 176 kb)

## Abbreviations

CHS: Conserved hairpin site
INT: Integrase
LTR: Long terminal repeats
PBS: Primer Binding site
RIP: Repeat-induced point mutation
RM: Repeat masker
RT: Reverse transcriptase
TEs: Transposable elements
