# Supplementary material for: Filamentous ascomycete genomes provide insights into Copia retrotransposon diversity in fungi
Source: BMC Genomics. 2017 May 25;18:410. doi: 10.1186/s12864-017-3795-2 (PMC5445492; doi:10.1186/s12864-017-3795-2)
Supplement: Supplementary file 2 — Phylogenetic relationships among fungal Copia families. Neighbor-Joining analysis of RT amino acid sequences of representative Copia families isolated with LTRharvest and all fungal Copia sequences available in RepBase. The 27 FunCo (Fungal Copia) clades are represented by their number in bold color. Statistical support (>70%) comes from non-parametric bootstrapping using 100 replicates. (PPTX 573 kb) [file 12864_2017_3795_MOESM2_ESM.pptx]

## Slide 1
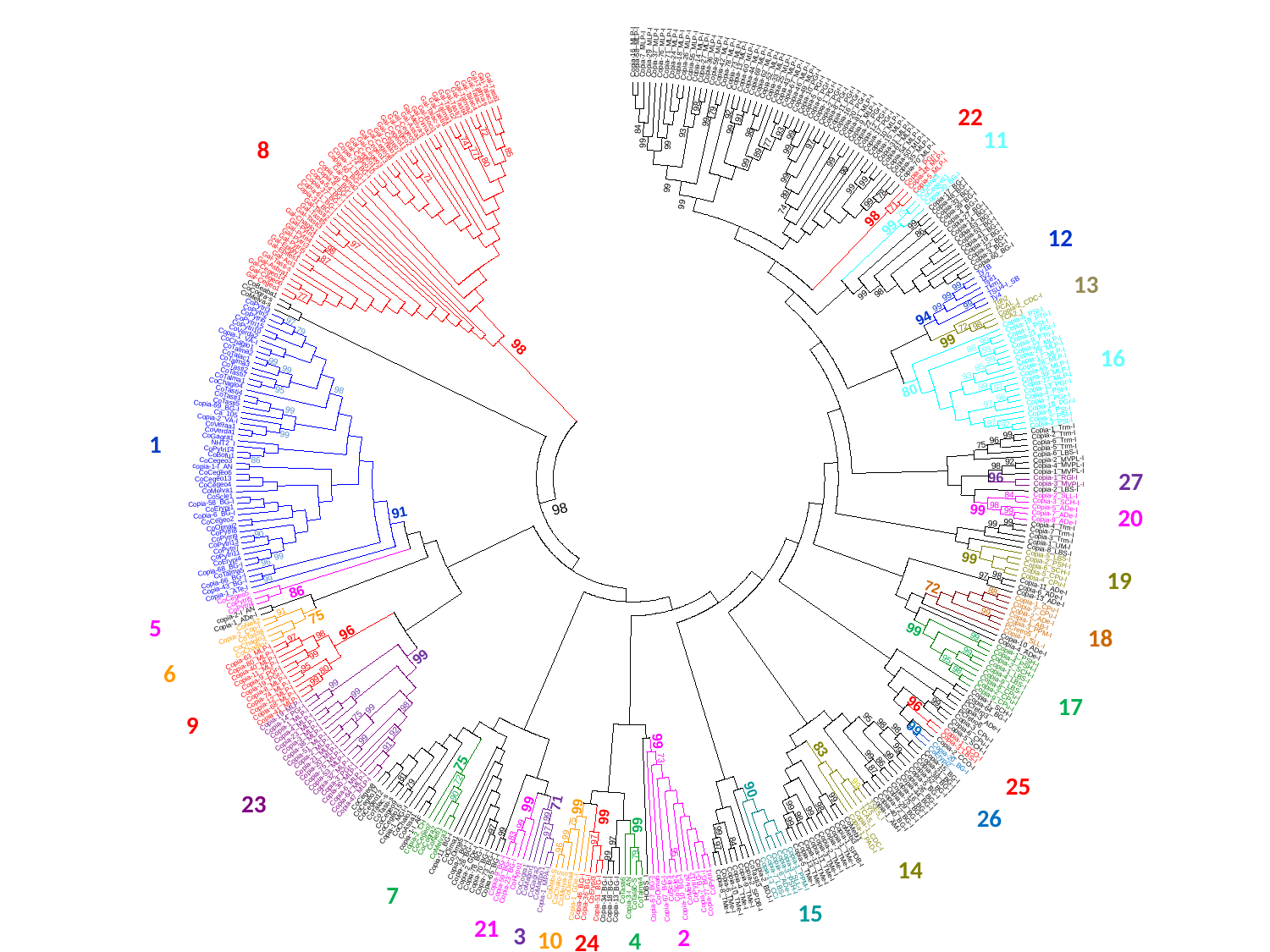

Copia-16_MLP-I
Copia-58_MLP-I
Copia-29_MLP-I
Copia-37_MLP-I
Copia-76_MLP-I
Copia-71_MLP-I
Copia-24_MLP-I
Copia-7_MLP-I
Copia-18_MLP-I
Copia-26_MLP-I
Copia-55_MLP-I
Copia-14_MLP-I
Copia-27_MLP-I
Copia-36_MLP-I
Copia-59_MLP-I
Copia-42_MLP-I
Copia-78_MLP-I
Copia-73_MLP-I
Copia-13_MLP-I
Copia-60_MLP-I
Copia-44_MLP-I
Copia-69_MLP-I
Copia-62_MLP-I
Copia-52_MLP-I
Copia-33_MLP-I
Copia-25_MLP-I
Copia-43_MLP-I
Copia-67_MLP-I
Copia-46_MLP-I
Gal-Tasti1
Gal-Talac3
Copia-49_MLP-I
Gal-Talma-s
Copia-10_PGr-I
Gal-Talac1
Gal-Talac4
Copia-5_PGr-I
Gal-Talma2
Copia-6_PGr-I
22
Copia-1_PGr-I
Gal-Tasti4
Gal-Tasti7
Copia-2_PGr-I
Gal-Talac2
98
Copia-8_PGr-I
Gal-Talma1
Copia-11_PGr-I
79
Copia-16_PGr-I
Gal-Talac-s
Gal-Tasti6
92
Copia-20_PGr-I
91
Copia-81_MLP-I
Gal-Boci1
Gal-Melva-s
Gal-Oima1
99
Copia-9_MLP-I
11
Copia-22_PGr-I
Gal-Assa1
Gal-Cegeo5
Copia-12_PGr-I
84
99
93
Gal-Cegeo2
72
Copia-17_PGr-I
98
8
93
Gal-Cegeo11
Copia-10_MLP-I
99
Copia-15_MLP-I
Gal-Cegeo7
74
77
Copia-31_MLP-I
Gal-Chalo1
99
97
99
Gal-Cegeo8
Copia-61_MLP-I
Gal-Cegeo13
99
Copia-54_MLP-I
77
85
89
Gal-Cegeo3
Copia-50_MLP-I
Gal-Cegeo12
Copia-35_MLP-I
Gal-Cegeo10
Copia-74_BG-I
99
Copia-70_MLP-I
80
99
Copia-71_BG-I
Copia-4_PGr-I
Copia-50_BG-I
99
Copia-48_MLP-I
Copia-5_MLP-I
99
71
Copia-49_BG-I
Gal-Oima2
99
Copia-8_BG-I
CoOima3
Copia-54_BG-I
CoCegeo11
99
99
Copia-30_BG-I
Copia-37_BG-I
Copia-17_BG-I
78
89
Copia-61_BG-I
Copia-48_BG-I
Copia-31_BG-I
99
Copia-33_BG-I
99
71
Copia-39_BG-I
74
Gal-Tasti2
75
Copia-4_BG-I
Gal-Tasti5
98
Copia-27_BG-I
Gal-Tasti3
12
Gal-Chaglo1
Copia-14_BG-I
99
99
Copia-63_BG-I
Gal-Pytri1
Copia-53_BG-I
86
Gal-Pytri4
Copia-41_BG-I
Gal-Pytri3
Copia-19_BG-I
Gal-Pytri5
97
Gal-Epigly1
Copia-22_BG-I
98
Gal-Epifes1
Copia-3_BG-I
Copia-60_BG-I
87
Gal-Aci1
Gal-Tasti-s
Gal-Asbra1
13
Ty1B
Gal-Cegeo14
Gal-Cegeo6
Ty2
Tse1
Gal-Cegeo1
Tkm1
99
TSU4-I_SB
CoBeaba1
98
CoCogra-s
99
99
Ty4
77
CoMelva-s
Tdh2
99
PCAL_I
Copia-2_CDC-I
CoPytri3
99
CoPytri2
94
TCA2_I
CoPytri6
Copia-6_PSt-I
97
CoPytri15
Copia-10_PTri-I
98
72
CoPytri10
Copia-1_PSt-I
79
CoVerda2
Copia-21_PGr-I
Copia-1_VA-I
99
99
Copia-9_PTri-I
16
CoChaglo1
98
Copia-22_MLP-I
88
93
CoTalma2
Copia-79_MLP-I
CoTalac1
Copia-17_MLP-I
99
99
CoTalma3
Copia-28_MLP-I
85
CoTasti2
Copia-65_MLP-I
99
CoTasti7
Copia-39_MLP-I
93
CoTalma1
Copia-77_MLP-I
CoChaglo4
99
97
Copia-13_PGr-I
80
95
98
CoTasti4
Copia-4_PSt-I
CoTasti1
96
Copia-7_PGr-I
CoTasti5
97
Copia-18_PGr-I
Copia-69_BG-I
99
Copia-5_PSt-I
Ca_105
Copia-2_PSt-I
Copia-2_VA-I
97
Copia-3_PSt-I
92
CoVeraa1
1
Copia-1_Trm-I
CoVerda1
99
99
Copia-2_Trm-I
CoGagra1
96
Copia-6_Trm-I
NHT2_I
75
Copia-5_Trm-I
CoPytri14
Copia-6_LBS-I
CoBofu1
Copia-2_MVPL-I
86
92
CoCegeo3
27
98
Copia-4_MVPL-I
copia-1-I_AN
Copia-1_MVPL-I
CoCegeo6
96
Copia-1_RGl-I
CoCegeo13
Copia-3_MVPL-I
CoCegeo4
Copia-2_LBS-I
CoMelva1
84
Copia-2_SLL-I
CoScle1
20
Copia-3_SCH-I
Copia-58_BG-I
98
98
99
91
Copia-5_ADe-I
CoErypi1
99
Copia-7_ADe-I
Copia-6_BG-I
CoCegeo2
Copia-9_ADe-I
99
99
Copia-4_Trm-I
CoOimai2
CoPytri8
Copia-7_Trm-I
90
CoPytri9
Copia-3_Trm-I
CoPytri13
Copia-1_UM-I
CoPytri7
Copia-8_LBS-I
99
99
CoPytri11
Copia-5_LBS-I
96
CoErypi4
Copia-2_PSH-I
19
Copia-6_SCH-I
Copia-68_BG-I
CoTalma5
98
Copia-5_CPu-I
97
99
Copia-4_CPu-I
Copia-66_BG-I
72
Copia-43_BG-I
Copia-11_ADe-I
86
88
Copia-6_ADe-I
Copia-1_ATe-I
CoCegeo5
Copia-13_ADe-I
CoPytri5
Copia-1_CPu-I
CoPytri4
91
5
Copia-7_CPu-I
99
75
copia-2-I_AN
Copia-2_ADe-I
18
Copia-1_AB-I
Copia-1_ADe-I
CoNefi2
99
96
Copia-3_PPM-I
PCretro5_I
98
Copia-2_Cop-I
99
CoTasti8
97
Copia-1_SLL-I
CoChaglo3
Copia-10_ADe-I
CoChaglo2
Copia-4_ADe-I
99
99
99
6
Copia-1_PSH-I
Copia-63_MLP-I
95
Copia-4_PSH-I
Copia-80_MLP-I
95
Copia-2_SCH-I
Copia-40_MLP-I
80
98
Copia-1_LBS-I
Copia-11_MLP-I
Copia-4_LBS-I
Copia-9_PGr-I
99
Copia-9_LBS-I
99
Copia-19_PGr-I
Copia-8_CPu-I
Copia-8_MLP-I
17
99
Copia-2_CPu-I
Copia-74_MLP-I
Copia-9_CPu-I
96
Copia-12_MLP-I
99
Copia-1_SCH-I
98
Copia-68_MLP-I
99
9
Copia-64_BG-I
PCretro3
Copia-41_MLP-I
75
Copia-19_MLP-I
95
PCretro6
Copia-3_ADe-I
Copia-14_PGr-I
98
Copia-1_MLP-I
99
98
Copia-4_MLP-I
Copia-3_CPu-I
92
Copia-6_CPu-I
Copia-23_MLP-I
99
Copia-3_MLP-I
99
Copia-5_SCH-I
Copia-38_MLP-I
Copia-1_CCO-I
91
83
99
Copia-3_LBS-I
Copia-51_MLP-I
Copia-2_MLP-I
Copia-2_CCO-I
99
99
73
CoErypi2_
Copia-20_BG-I
Copia-21_MLP-I
75
86
Copia-20_MLP-I
Copia-75_MLP-I
Copia-15_BG-I
87
25
Copia-21_BG-I
Copia-53_MLP-I
Copia-38_BG-I
81
Copia-32_MLP-I
Copia-3_PGr-I
72
Copia-52_BG-I
99
Copia-5_BG-I
79
Copia-30_MLP-I
90
23
Copia-6_MLP-I
Copia-26_BG-I
Copia-44_BG-I
Copia-64_MLP-I
90
CoCegeo8
Copia-16_BG-I
99
Copia-47_MLP-I
71
99
Copia-75_BG-I
26
CoCegeo17
99
Copia-29_BG-I
99
CoCegeo12
98
CoTalac-s
Copia-72_BG-I
CoTasti-s
TY5
99
TSE5_I
Copia-40_BG-I
99
CoCegeo9
Copia-1_AM-I
98
99
TCA5_I
CoCegeo15
99
75
99
CoChalo1
99
Copia-1_MGI-I
CoAssa1
97
CoMebi1
99
Copia-1_CDC-I
97
99
CoMebi3
copia-1-I_AF
Copia-1_PAD-I
99
83
Copia-2_CH
CoCegeo7
CoNefi1
97
97
CoCogra2
84
CoMelva3
Copia-1_SPDB-I
97
Copia-1_TMe-I
96
CoOima1
Copia-7_TMe-I
CoOima5
95
14
Copia-2_TMe-I
Copia-11_BG-I
79
99
Copia-3_TMe-I
Copia-2_BG-I
Copia-11_TMe-I
Copia-12_TMe-I
Copia-28_BG-I
Copia-13_TMe-I
Copia-5_TMe-I
Copia-2_GDe-I
Copia-1_PPM-I
Copia-78_BG-I
Copia-3_SLL-I
Copia-70_BG-I
Copia-3_PSH-I
Copia-73_BG-I
CoTasti3
Copia-8_ADe-I
Copia-65_BG-I
Copia-9_BG-I
CoHypo1
Copia-7_LBS-I
7
Copia-1-I_CCi
Copia-59_BG-I
CoCogra1
CoMagor1
Copia-2_BDJ-I
Copia-23_BG-I
CoPytri1
CoGagra2
CoMagpo1
CoMebi-S
Copia-9_TMe-I
CoOima4
Copia-2_SPDB-I
CoPytri12
HOBS_I
Copia-4_TMe-I
CoChalo-S
CoScle2
CoScle3
CoMelva-S
CoMelva2
CoErypi3
CoOima6
Copia-1_MPA-I
CoTasti6
Copia-6_TMe-I
Copia-10_TMe-I
Copia-7_BG-I
Copia-8_TMe-I
CoTalma4
Copia-77_BG-I
CoTalac-S
15
Copia-46_BG-I
Copia-36_BG-I
Copia-10_BG-I
Copia-1_GDe-I
Copia-3-I_AN
Copia-51_BG-I
Copia-67_BG-I
Copia-57_BG-I
Copia-34_BG-I
Copia-18_BG-I
Copia-13_BG-I
21
3
2
10
4
24
