# Supplementary material for: Filamentous ascomycete genomes provide insights into Copia retrotransposon diversity in fungi
Source: BMC Genomics. 2017 May 25;18:410. doi: 10.1186/s12864-017-3795-2 (PMC5445492; doi:10.1186/s12864-017-3795-2)
Supplement: Supplementary file 6 — Charaterization of the extended Conserved Hairpin Site. (A) Local alignment of Tasti2, Tasti4 and Tasti5 sequences from Talaromyces stipitatus showing the different regions of the extended Conserved Hairpin Site (CHS). (B) Families in which different extended CHS were observed. For each extended CHS observed, the corresponding size (in bp) of its variable regions is given. (PPTX 126 kb) [file 12864_2017_3795_MOESM6_ESM.pptx]

## Slide 1
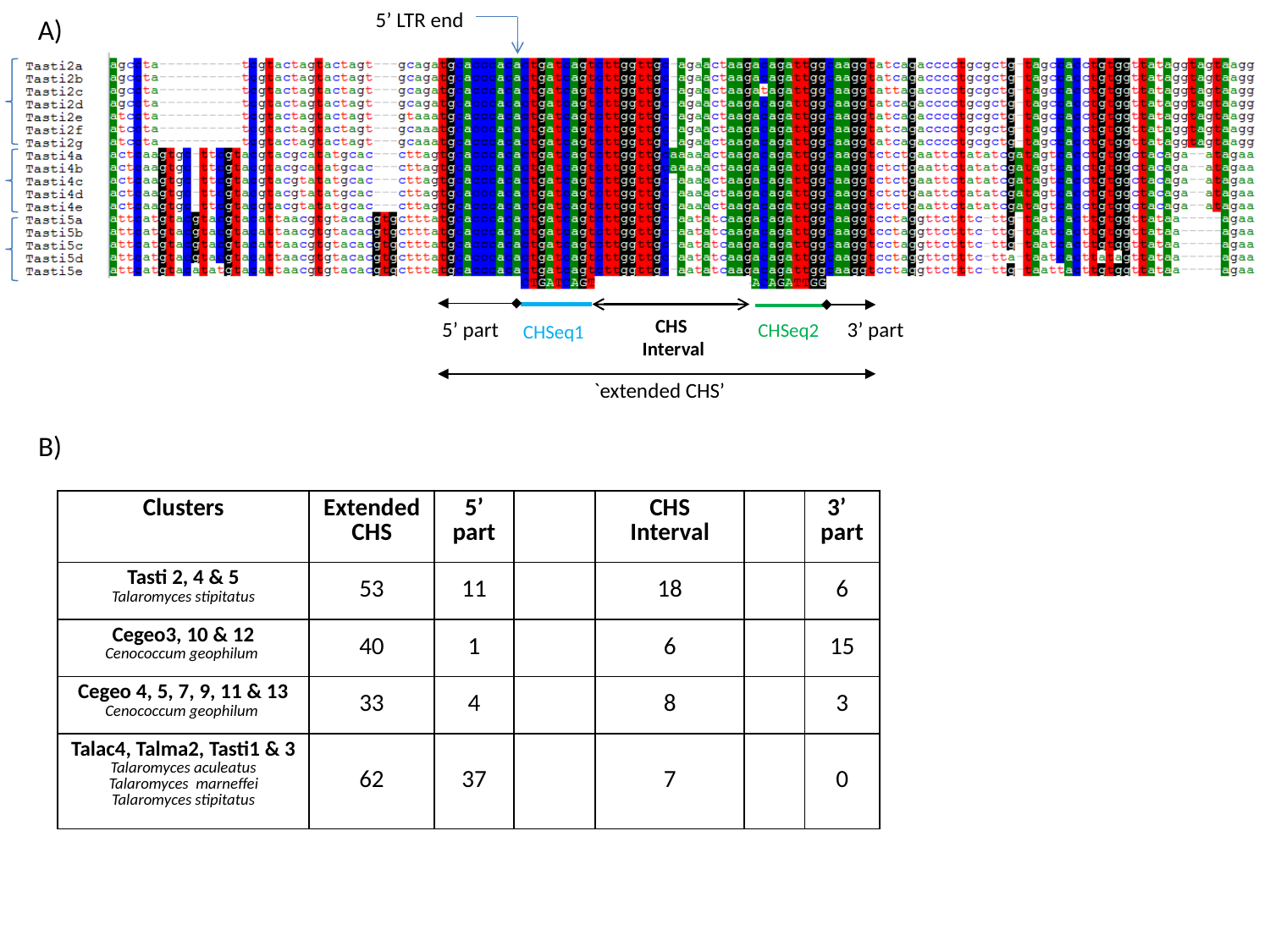

5’ LTR end
A)
CHS
Interval
5’ part
3’ part
CHSeq2
CHSeq1
`extended CHS’
B)
| Clusters | Extended CHS | 5’ part | | CHS Interval | | 3’ part |
| --- | --- | --- | --- | --- | --- | --- |
| Tasti 2, 4 & 5 Talaromyces stipitatus | 53 | 11 | | 18 | | 6 |
| Cegeo3, 10 & 12 Cenococcum geophilum | 40 | 1 | | 6 | | 15 |
| Cegeo 4, 5, 7, 9, 11 & 13 Cenococcum geophilum | 33 | 4 | | 8 | | 3 |
| Talac4, Talma2, Tasti1 & 3 Talaromyces aculeatus Talaromyces marneffei Talaromyces stipitatus | 62 | 37 | | 7 | | 0 |
