# Supplementary figures and images for: Filamentous ascomycete genomes provide insights into Copia retrotransposon diversity in fungi
Source: BMC Genomics. 2017 May 25;18:410. doi: 10.1186/s12864-017-3795-2 (PMC5445492; doi:10.1186/s12864-017-3795-2)

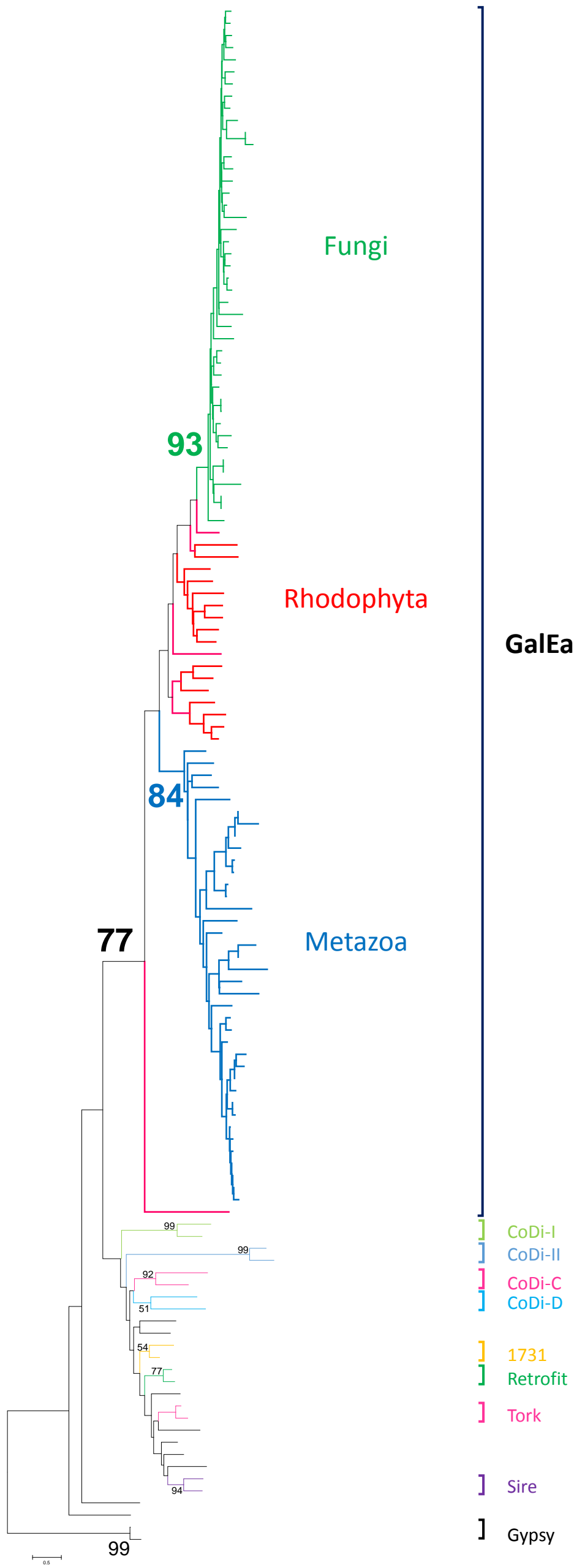

Supplement: Supplementary file 7 — Phylogenetic relationships among GalEa retrotransposons. Neighbor-Joining analysis of RT/RNaseH amino acid sequences of GalEa elements and representative Copia clades previously defined in the Gypsy Database. Statistical support comes from non-parametric bootstrapping using 100 replicates. (PDF 178 kb) [file 12864_2017_3795_MOESM7_ESM.pdf]

A)

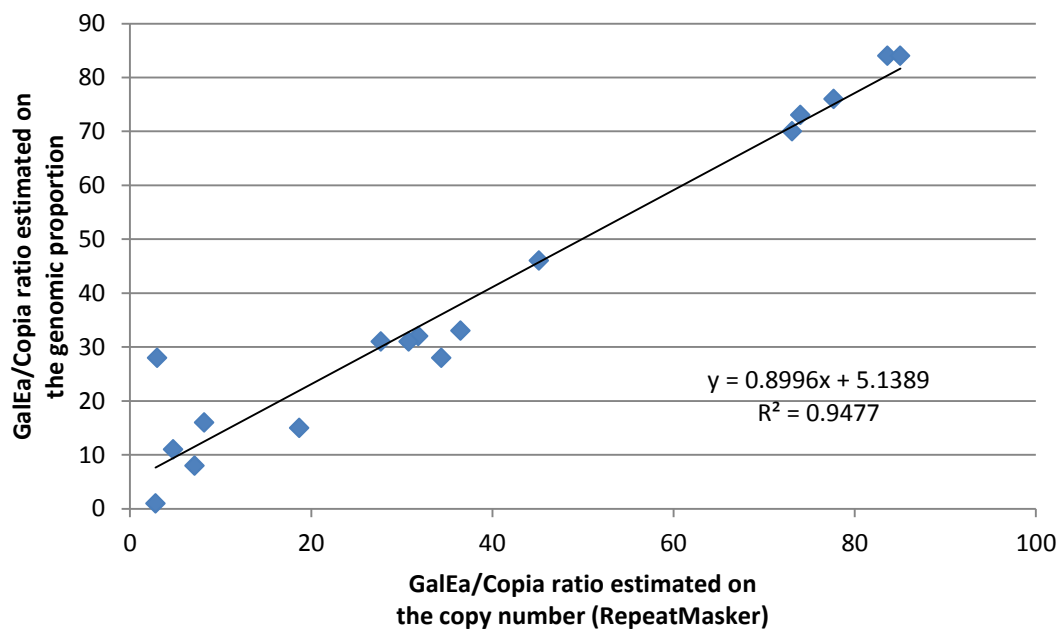

B)

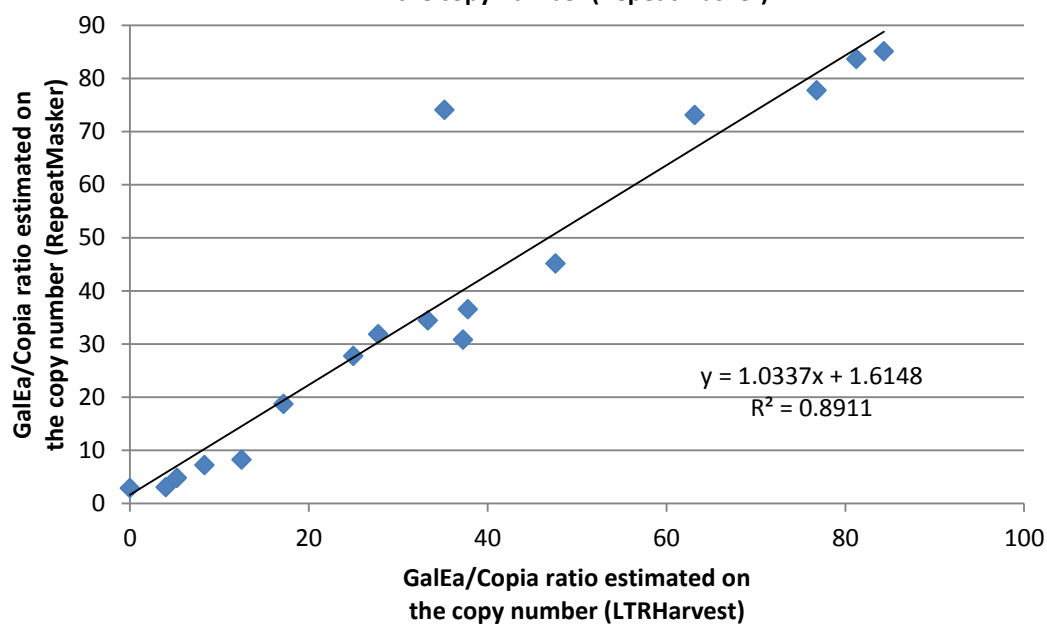

C)

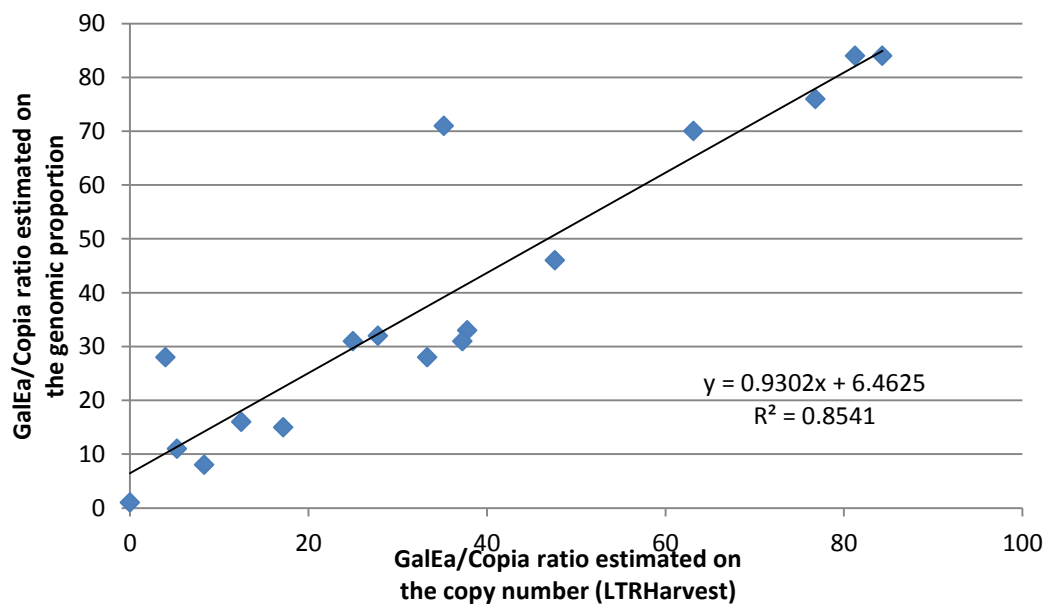

Supplement: Supplementary file 8 — Comparison of methods used to estimate the proportion of GalEa elements among fungal Copia retrotransposons. We compared in pairs the three estimations of GalEa proportions among Copia based on the number of copies detected with LTRharvest, the number of large copies detected with RepeatMasker, or the genomic proportions derived from RepeatMasker for the 17 genomes that harbor at least 10 Copia sequences detected with LTRharvest. (PDF 176 kb) [file 12864_2017_3795_MOESM8_ESM.pdf]
